# Using selenomethionyl derivatives to assign sequence in low-resolution structures of the AP2 clathrin adaptor

**DOI:** 10.1107/S2059798315021580

**Published:** 2016-03-01

**Authors:** Bernard T. Kelly, Stephen C. Graham, David J. Owen

**Affiliations:** a Cambridge Institute for Medical Research, Department of Clinical Biochemistry, University of Cambridge, Hills Road, Cambridge CB2 0XY, England; b Department of Pathology, University of Cambridge, Tennis Court Road, Cambridge CB2 1QP, England

**Keywords:** selenomethionyl derivatives, sequence assignment, low resolution, AP2 clathrin adaptor complex

## Abstract

Selenomethionine incorporation is a powerful technique for assigning sequence to regions of electron density at low resolution. Genetic introduction of methionine point mutations and the subsequent preparation and crystallization of selenomethionyl derivatives permits unambiguous sequence assignment by enabling the placement of the anomalous scatterers (Se atoms) thus introduced. Here, the use of this approach in the assignment of sequence in a part of the AP2 clathrin adaptor complex that is responsible for clathrin binding is described. AP2 plays a pivotal role in clathrin-mediated endocytosis, a tightly regulated process in which cell-surface transmembrane proteins are internalized from the plasma membrane by incorporation into lipid-enclosed transport vesicles. AP2 binds cargo destined for internalization and recruits clathrin, a large trimeric protein that helps to deform the membrane to produce the transport vesicle. By selenomethionine labelling of point mutants, it was shown that the clathrin-binding site is buried within a deep cleft of the AP2 complex. A membrane-stimulated conformational change in AP2 releases the clathrin-binding site from autoinhibition, thereby linking clathrin recruitment to membrane localization.

## Abstract 2

A selenomethionine marker strategy allowed the identification of a region of disconnected electron density at low resolution and despite poor selenomethionine incorporation, thereby building a structural framework for understanding how the clathrin adaptor AP2 regulates clathrin binding in mammalian cells.

## 1. Introduction   

Eukaryotic cells contain a plethora of specialized lipid membrane-enclosed organelles. Transmembrane proteins (and often their luminal cargo) are transported between these organelles in a controlled fashion to ensure the correct functioning of the cell. For example, activated cell surface receptors are often downregulated by internalization from the plasma membrane and delivery to lysosomes, where they are degraded. Transmembrane-protein ‘cargo’ is moved between organelles by incorporation into small membrane-bound transport containers termed ‘vesicles’ that bud off one organelle and are transported to and fuse with a second (destination) organelle. This process has to be tightly regulated to ensure that proteins are delivered in a timely and accurate manner. Thus, eukaryotes have evolved a modular trafficking system in which transmembrane proteins and organelles are marked with signals that interact with the cytosolic proteins that control inter-organelle traffic (Traub, 2009 ▸).

Cell surface receptors are often marked by the presence of short, linear amino-acid trafficking motifs; likewise, the internal leaflet of the plasma membrane is itself marked by the presence of the phosphoinositide PtdIns(4,5)P_2_. Specialized trafficking adaptor proteins termed ‘clathrin adaptors’, targeted to the plasma membrane by interactions with PtdIns(4,5)P_2_, recognize and bind these trafficking motifs whilst simultaneously recruiting the large trimeric protein clathrin (Owen *et al.*, 2004 ▸). In this way, clathrin adaptors sequester transmembrane-protein cargo destined for removal from the plasma membrane into nascent bud-like structures coated with clathrin, which polymerizes and drives the formation of a curved bud or ‘pit’. Ultimately, the clathrin-coated bud is pinched off the membrane for delivery to internal compartments.

**Figure 1:**
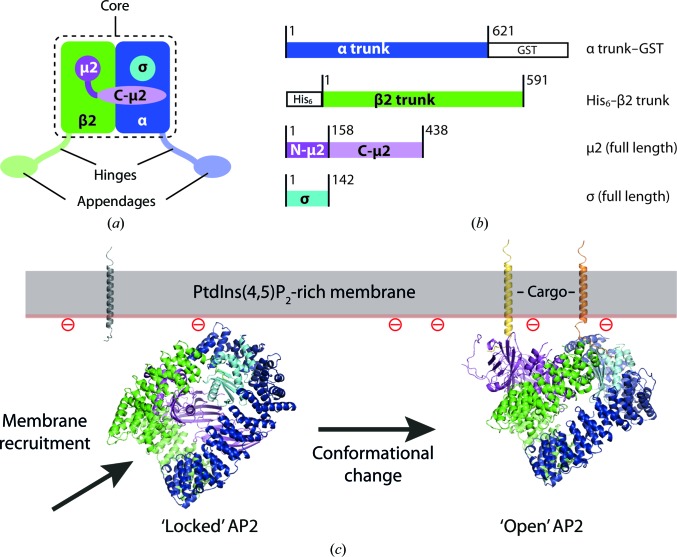
(*a*) Schematic representation of the AP2 clathrin adaptor. The ‘core’ is indicated; the α and β2 hinge and appendage subdomains are shown in paler colours to indicate the fact that they do not form part of the core. Note that μ2 is composed of an N-terminal longin-fold subdomain and a C-terminal ‘μ-homology’ subdomain. (*b*) Schematics of the AP2 constructs used to express recombinant AP2 core in *E. coli*, showing the C-terminally GST-tagged α trunk subdomain, the N-terminally hexahistidine-tagged β2 trunk subdomain and the μ2 and σ2 subunits. (*c*) Conformational states of the AP2 core. ‘Locked’ AP2 (PDB entry 2vgl; Collins *et al.*, 2002 ▸) is unable to bind cargo owing to steric blockage of the binding sites by the β2 subunit. Upon plasma-membrane recruitment [driven by association with PtdIns(4,5)P_2_], the complex undergoes a conformational change that reveals the cargo-binding sites, allowing cargo recruitment and stabilization of the ‘open’ conformation (PDB entry 2xa7; Jackson *et al.*, 2010 ▸).

The major clathrin adaptor present at the plasma membrane is the assembly polypeptide 2 (AP2) complex. AP2 binds two commonly found internalization motifs [Y*xx*φ, where φ denotes a bulky hydrophic residue, and (DE)*xxx*L(LI); Bonifacino & Traub, 2003 ▸]. AP2 is a large (∼300 kDa) heterotetrameric complex comprising large α and β2 subunits, a medium μ2 subunit and a small σ2 subunit. The N-terminal ‘trunk’ regions of α and β2, together with μ2 and σ2, form the globular ‘core’ of the complex (Collins *et al.*, 2002 ▸; Fig. 1 ▸ *a*) that binds both the Y*xx*φ and (DE)*xxx*L(LI) motifs and PtdIns(4,5)P_2_. The C-terminal ‘appendage’ domains of α and β2 are separated from the core by flexible (largely unstructured) ‘hinges’ (Heuser & Keen, 1988 ▸). Structural and biochemical studies of the AP2 core in our laboratory employ AP2 expressed in *Escherichia coli* from a pair of plasmids, one encoding a C-terminally GST-tagged α trunk domain and the whole σ2 subunit and the other encoding an N-terminally hexahistidine-tagged β2 trunk domain and the whole of the μ2 subunit (Fig. 1 ▸ *b*), and purified sequentially by GSH and Ni–NTA affinity chromatography (Collins *et al.*, 2002 ▸).

The AP2 core exists in two different conformational states (Jackson *et al.*, 2010 ▸; Fig. 1 ▸ *c*). In the ‘locked’ or inactive, cytosolic state (Fig. 1 ▸ *c*, left) the internalization motif-binding sites are blocked by residues of the β2 subunit and the PtdIns(4,5)P_2_-binding sites are on different faces of the complex. In the ‘open’ or active state all of the known ligand-binding sites are coplanar (Jackson *et al.*, 2010 ▸; Fig. 1 ▸ *c*, right). The conformational change from the locked to the open state is driven by binding to membranes containing PtdIns(4,5)P_2_ and stabilized by binding to cargo that contains the correct internalization motifs. Thus, AP2 acts as a membrane-activated switch (driven by coincidence detection) that prevents cargo recognition except at the plasma membrane.

AP2 also binds and recruits clathrin to sites of coated pit initiation. Clathrin binds AP2 at two sites: a short ‘clathrin-box’ motif (LLNLD) in the unstructured hinge of β2 binds the clathrin N-terminal β-propeller domain and a second site on the C-terminal β2 appendage sub­domain binds the clathrin ‘leg’, although binding at this second site is significantly weaker (Owen *et al.*, 2000 ▸). Biochemical evidence in our laboratory suggested that clathrin binding by AP2 is regulated similarly to cargo binding, such that clathrin recruitment was stimulated by the simultaneous binding of membrane-localized PtdIns(4,5)P_2_ and cargo, whereas clathrin binding was poor in the absence of such signals (Kelly *et al.*, 2014 ▸).

## 2. Crystallization of an extended AP2 core   

**Figure 2:**
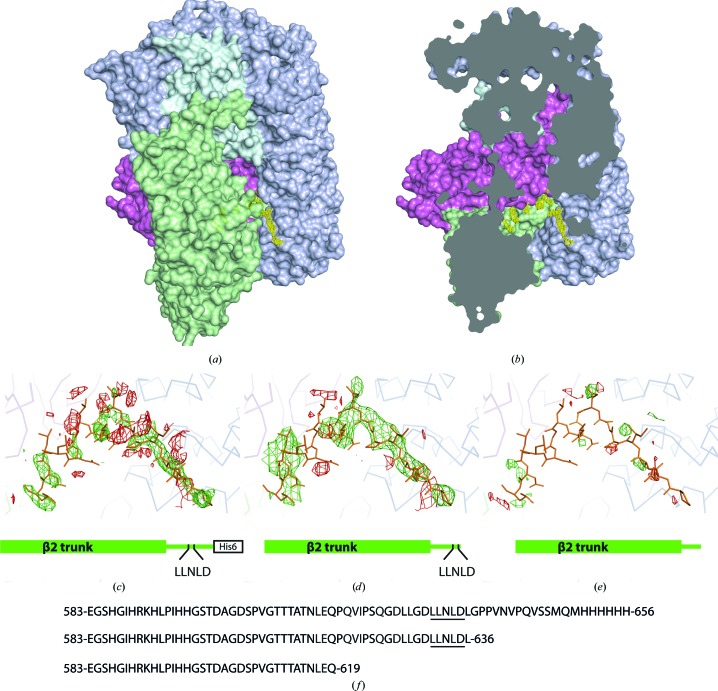
(*a*) Surface representation of the βhingeHis6.AP2 ‘extended core’ complex, coloured as in Fig. 1 ▸ but in paler shades for clarity. Shown in yellow is the positive *mF*_o_ − *DF*_c_ difference density in the ‘bowl’ after restrained refinement of the locked AP2 core structure lacking the hinge fragment (PDB entry 2vgl) against the βhingeHis6.AP2 data. (*b*) Cutaway of the same view of the complex as in (*a*), showing the buried difference density deep in the ‘bowl’. (*c*–*e*) *mF*_o_ − *DF*_c_ maps (contoured at ±3σ, positive density in green and negative density in red) after restrained refinement of the locked AP2 core structure (PDB entry 2vgl) against the βhingeHis6.AP2 data (*c*) and against data obtained from constructs with β2 subunits truncated at Leu636 (*d*) and Gln619 (*e*). The appropriate β2 subunit is shown schematically beneath each map. (*f*) C-terminal sequences of the β2 constructs depicted in (*c*), (*d*) and (*e*), starting at the first disordered residue of the β2 hinge region and with the clathrin-box motif underlined.

To investigate this observation, we attempted to crystallize a form of the AP2 complex comprising the whole of the β2 subunit (and thus both clathrin-interacting sites) along with the μ2 and σ2 subunits and the trunk sub­domain of α. Unfortunately, we were unable to crystallize this complex. Next, we constructed a version of the AP2 core complex extended to include a 68-residue part of the unstructured β2 hinge (including the clathrin-box motif; Fig. 2 ▸ *f*), which we termed βhingeHis6.AP2. The extended β2 subunit in our βhingeHis6.AP2 construct ended at Met650, whereas the β2 subunit that we had previously used to determine the core structure ended at residue Lys591 and the last ordered residue discernible in the core structure was Val582. We were concerned that the unstructured segment of the β2 hinge might be prone to proteolysis. We therefore moved the hexahistidine tag to the C-terminus of β2 in an attempt to ensure that only AP2 complexes containing full-length β2 (*i.e.* trunk plus hinge fragment) are bound during the Ni–NTA purification step. This extended β2 subunit construct was successfully crystallized in the same conditions that were previously used to grow crystals of the AP2 core in the locked (inactive) conformational state (Collins *et al.*, 2002 ▸).

Crystals were grown at 16°C from a mixture of 15 mg ml^−1^ AP2 with 1 mg ml^−1^ IP6 [d-*myo*-inositol 1,2,3,4,5,6-hexakis­phosphate; Calbiochem; an analogue of PtdIns(4,5)P_2_] by hanging-drop vapour diffusion against a reservoir consisting of 18% PEG 1000, 100 m*M* sodium/potassium phosphate pH 6.2, 200 m*M* NaCl, 4 m*M* DTT. Crystals were cryoprotected in mother liquor augmented with 20% glycerol and 1 mg ml^−1^ IP6 and cryocooled by plunging into liquid N_2_. Data were collected at 100 K on beamline I03 at Diamond Light Source (DLS). The crystals belonged to space group *P*3_1_21, with unit-cell parameters *a* = 122, *c* = 259 Å, and typically diffracted to around 2.8 Å resolution overall. Data were integrated, scaled and merged with *XDS*, *XSCALE* (Kabsch, 2010 ▸) and *SCALA* (Evans, 2006 ▸) using the automated data-processing package *xia*2 (Winter, 2010 ▸). A summary of the crystallographic data is presented in Table 1 ▸.

Since the crystals were isomorphous to crystals of the AP2 core in the locked conformation, the structure of the locked AP2 core (PDB entry 2vgl; Collins *et al.*, 2002 ▸) was refined against the new data with *REFMAC*5 (Murshudov *et al.*, 2011 ▸) using TLS and restrained refinement. When AP2 transitions from the locked to the open conformation, the alpha solenoids of the α and β2 trunk subdomains flex around several hinge points (Jackson *et al.*, 2010 ▸), defining a set of rigid subdomains that we used as TLS groups in the refinement of the new AP2 locked-core structure. After initial refinement, a difference electron-density (*mF*_o_ − *DF*_c_) map suggested additional, unmodelled electron density buried in a deep cleft of the core that we term the ‘bowl’ of AP2 (Figs. 2 ▸ *a* and 2 ▸ *b*). This region of electron density was disconnected from the rest of the core and we were unable to discern side chains that might allow us to positively identify the buried residues. Given the presence of the clathrin-box motif in the segment of the β2 hinge included in our extended AP2 construct, and given our biochemical observations suggesting that the locked conformation of AP2 was unable to bind clathrin efficiently, it became important to determine exactly which part of the hinge was buried in the bowl. If it transpired that the clathrin-box motif itself, or a closely flanking region, was buried in the bowl then this would provide a plausible structural mechanism to reduce inappropriate clathrin binding and, potentially, link clathrin recruitment to a membrane-stimulated conformational change.

## 3. Truncation-mutant analysis   

To begin to narrow down the buried region, we constructed mutants of βhingeHis6.AP2 truncated after Gln619 and Leu636 (Fig. 2 ▸ *f*) and lacking the C-terminal hexahistidine tag to avoid the possibility of the tag interfering with binding in the bowl. These mutants were expressed and crystallized as described above, yielding crystals that were isomorphous to those of the nontruncated complex. Refinement of the AP2 core complex structure against these data revealed that the Leu636 truncation mutant retained the unmodelled difference density in the bowl (Fig. 2 ▸ *d*), whereas the Gln619 truncation mutant did not (Fig. 2 ▸ *e*). This suggested that the buried sequence was N-terminal to Leu636 and might lie between Gln619 and Leu636. On this basis, we prepared preliminary models that placed the region between residues 619 and 636 into the difference electron density visible in the bowl. Secondary-structure prediction using the *JPred* server (Cole *et al.*, 2008 ▸) suggested the presence of a short region of helix spanning Asp626–Leu631. At low contour levels, a 2*mF*_o_ − *DF*_c_ map hinted at a possible helical region in the buried electron density; as a result, our first model was built on this basis. The occupancy of the buried fragment when refined with fixed *B* factors in *REFMAC*5 was ∼0.8. We then prepared a series of models sequentially shifted by one residue at a time. The quality of the electron density was, however, insufficient to differentiate between these models. Similarly to all AP2 structures determined to date, the β2 subunit is less well ordered than the σ subunit or the N-terminal regions of the α subunit abutting σ, probably because β2 acts as a ‘latch’ to hold the complex shut and is thus poised to swing away from σ and μ2 in order to reveal the cargo-binding sites (Jackson *et al.*, 2010 ▸). It is therefore not surprising that the buried portion of the β2 hinge is likewise comparatively poorly ordered, rendering definitive identification of the residues problematic. It remained possible that the buried region lay partly or wholly N-terminal to Gln619 and that the removal of residues 619–636 destabilized the hinge–bowl interaction perhaps owing to a loss of weaker, secondary interactions. Thus, we sought a way to identify the buried residues definitively.

## 4. Analysis of (seleno)methionine point mutants   

**Figure 3:**
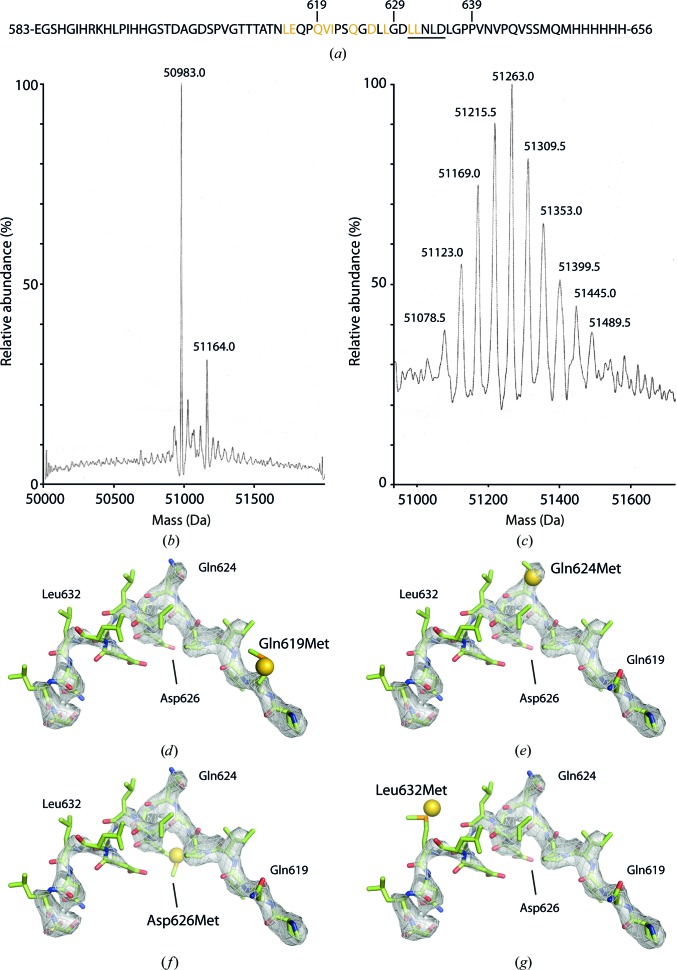
(*a*) Residues (highlighted in gold) chosen for single-site mutagenesis to methionine in the β2 hinge. The clathrin-box motif is underlined. (*b*, *c*) Mass spectra of the μ2 subunit of native (*a*) and selenomethionyl (*b*) ‘wild-type’ βhingeHis6.AP2. The masses of major peaks are shown. (*d*–*g*) Final 2*mF*_o_ − *DF*_c_ maps in the hinge region (contoured at 0.34 e^−^ Å^−3^) with overlaid hinge models showing residues mutated to methionine along with selenium sites (shown as gold balls) found in the appropriate data sets, indicating good agreement between the sites and the hinge model.

Others have successfully used methionine point mutants incorporating selenomethionine (SeMet) to identify regions of structure in low-resolution maps (Pomeranz Krummel *et al.*, 2009 ▸; Oubridge *et al.*, 2009 ▸) or for chain tracing (Evans, 2003 ▸). We decided to pursue a similar strategy to identify the residues buried in the bowl of AP2. Apart from two methionine residues at the extreme C-terminus, the β2 hinge fragment in our construct lacks endogenous methionines (Fig. 2 ▸ *f*). We therefore constructed a series of point mutants in which single residues were substituted with methionine (Fig. 3 ▸ *a*). Initially, we chose hydrophobic residues (valine, isoleucine and leucine) together with glutamine or glutamate residues (which contain an aliphatic side chain similar in length to methionine) to mutate. We subsequently mutated a single aspartate in order to bridge a gap of three residues between neighbouring mutation sites. By crystallizing each mutant and pinpointing selenium sites, we hoped to determine the position of the single introduced methionine in each case and thereby trace the residues buried in the bowl.

Initial attempts to express ‘wild-type’ βhingeHis6.AP2 using a methionine-auxotroph strain (B834) grown in minimal medium supplemented with selenomethionine were unsuccessful. We therefore attempted to employ a methionine-biosynthesis pathway inhibition approach (Van Duyne *et al.*, 1993 ▸). In this technique, the endogenous *E. coli* methionine-biosynthetic pathway is suppressed by the addition of a cocktail of amino acids that cause product inhibition of key enzymes in the pathway, while the minimal growth medium is supplemented with selenomethionine. This approach also initially failed to produce AP2. We next attempted to ‘kick-start’ the expression of AP2 by supplementing the minimal growth medium with some rich broth (∼25%). This approach proved quite successful insofar as crystallographically useful quantities of purified AP2 were produced. To estimate the efficiency of selenomethionine incorporation, we analyzed both native and selenomethionyl ‘wild-type’ βhingeHis6.AP2 by electrospray mass spectrometry using a Waters Micromass LCT (Figs. 3 ▸ *b* and 3 ▸ *c*). With the native protein, a strong peak was found at approximately the expected molecular weight of the μ2 subunit (expected mass 50 971 Da; observed mass 50 983 Da; Fig. 3 ▸ *b*); a peak was also found for the σ2 subunit, although the mass spectrum was noisier. Strong peaks corresponding to the α and β2 subunits were not found; however, since all four subunits are coexpressed in the same bacterial cells, we assume that the labelling efficiency for μ2 is representative of the whole complex. With the selenomethionyl protein, mass spectrometry revealed a series of peaks approximately normally distributed around a central peak corresponding to a μ2 subunit with a mass of 51 263 Da (280 Da greater than the native protein; Fig. 3 ▸ *c*). The mean separation between the peaks is 45.7 Da, which is close to the expected difference in molecular mass between sulfur and selenium (∼47 Da); thus, the observed peaks correspond to proteins differing by a single substituted selenomethionine. The 280 Da difference in mass between the main peaks of the native and selenomethionyl proteins suggests an incorporation efficiency of ∼45% in the 14 methionine residues of μ2. We therefore estimated the overall incorporation efficiency to be 45%.

Selenomethionyl derivatives of wild-type and mutant βhingeHis6.AP2 were produced and crystallized. Two mutants failed to crystallize in initial attempts (E616M and I621M) and were not investigated further. Most crystallized in the same conditions and with the same space group and unit-cell parameters as the wild-type βhingeHis6.AP2 complex. In practice, we find that crystals of AP2 vary greatly in the extent of their diffraction despite uniformity of gross morphology, and individual large AP2 crystals may diffract non-uniformly across the crystal volume. Thus, we routinely screened both multiple crystals and multiple positions within larger single crystals to maximize our chances of obtaining the best possible diffraction data. In the case of the selenomethionyl derivatives the best crystals diffracted to around 3 Å resolution. Data were collected at a wavelength of ∼0.98 Å (∼0.91 Å in some cases owing to the constraints of beamline availability) on beamlines I02, I03 and I04-1 at DLS. In all cases we sought to maximize anomalous multiplicity in order to improve the accuracy in measurement of anomalous differences, whilst avoiding excessive radiation damage. Data sets were collected from crystals diffracting to better than ∼3.5 Å resolution. We collected multiple data sets for each mutant (typically three, but ranging from one to eight). Data were integrated, scaled and merged with *XDS*, *XSCALE* (Kabsch, 2010 ▸) and *AIMLESS* (Evans & Murshudov, 2013 ▸), using the automated data-processing package *xia*2 (Winter, 2010 ▸) and custom scripts to automate processing of all selenomethionyl mutant data sets. In some cases, two data sets were merged in order to improve the accuracy of anomalous signal measurement (Liu *et al.*, 2011 ▸). Consistent indexing was enforced by specifying a reference data set in *xia*2. This was necessary because there are two valid axis definitions in *P*3_1_21 (related by the operator −*h*, −*k*, *l*).

## 5. Location of selenium sites by anomalous log-likelihood gradient map completion   

**Figure 4:**
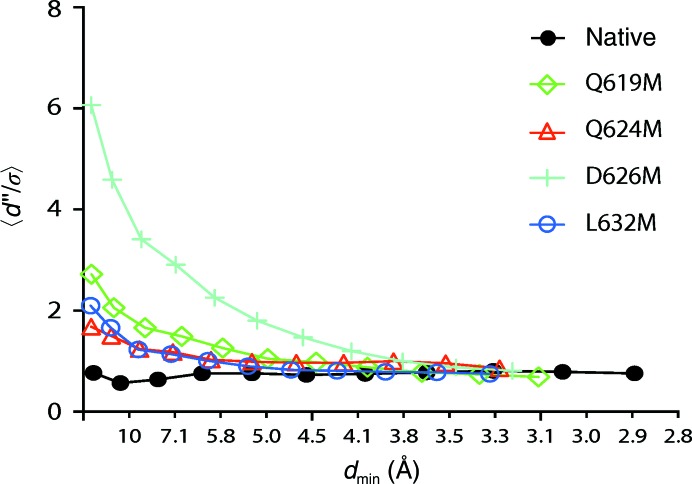
Anomalous differences as a function of resolution for native and four selenomethionyl mutant data sets calculated by *SHELXC*. The mean ratio of anomalous differences to their estimated standard deviation (*d*′′/σ; vertical axis) is plotted against *d*_min_ (resolution at the midpoint of the bin; horizontal axis). Except for the D626M data set, significant *d*′′/σ (above ∼1.3; Schneider & Sheldrick, 2002 ▸) is not present beyond ∼6 Å resolution. The Q619M and D626M data sets are two-crystal merged data sets; Q624M and L632M are single data sets. Note that the D626M data set, which displays noticeably better anomalous signal, was the only one of these data sets for which the anomalous substructure could be solved using *SHELXD*.

In almost all cases the anomalous signal was quite weak, with useful signal generally not extending beyond ∼6 Å resolution (as judged by the resolution at which the ratio of anomalous differences to their estimated standard deviations drops below ∼1.3; Schneider & Sheldrick, 2002 ▸; Fig. 4 ▸). In the absence of any other phase information, this would make substructure solution difficult, and indeed attempts to solve the substructure with *SHELXD* (Schneider & Sheldrick, 2002 ▸) failed with all but one of the mutant data sets (D626M). Given the low incorporation of selenomethionine (∼45%) and the large number of selenomethionine sites (38 in the core), this is not surprising. However, our goal was not to solve the structure using experimental phases, but rather to identify selenium marker sites. Therefore, we could make use of this weak anomalous data to find sites by using phases calculated from our existing AP2 locked-core model. Our strategy was to identify anomalous scatterers (*i.e.* selenium sites) by iterative substructure completion using anomalous log-likelihood gradient maps with *Phaser-EP*, where starting phases were provided by an AP2 model refined against the new data and including a ‘best-guess’ model of the buried hinge fragment. In this approach, SAD log-likelihood gradient maps are searched for sites where the addition of an anomalous scatterer would improve the fit of the anomalous scattering model to the experimental data and, after new sites have been identified, the process is iterated until the map is ‘flat’ (Read & McCoy, 2011 ▸). The likelihood formulation has the advantage of increased sensitivity compared with simple difference Fouriers (de La Fortelle & Bricogne, 1997 ▸). We used custom scripts to automate the substructure completion with *Phaser-EP*. The *Z*-score cutoff for addition of new sites was set at the default level of 6.

**Figure 5:**
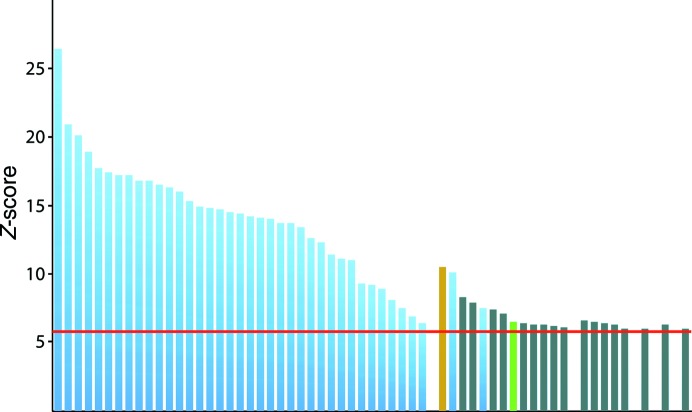
Anomalous scattering sites located by *Phaser-EP* from two merged Q619M data sets in successive cycles of map completion (cycles indicated by gaps between sets), showing *Z*-scores (vertical axis) for each site. Blue bars indicate ‘core’ methionines, the gold bar indicates the hinge site ascribed to the methionine point mutant (Q619M), grey bars indicate ‘core’ methionine alternative conformations and the green bar was interpreted as a chloride ion.

**Figure 6:**
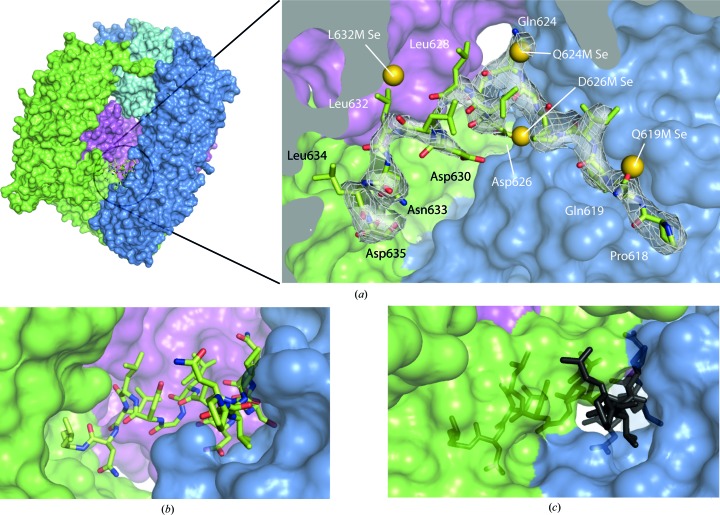
(*a*) Overall (left) and close-up (right) views of the structure of βhingeHis6.AP2 (PDB entry 4uqi). The residues of the hinge resolved in the structure are shown in green as a stick representation. The AP2 core is depicted as a surface representation coloured as in Fig. 1 ▸. The residues of the buried hinge are indicated in the close-up view, with electron density shown as a mesh (2*mF*_o_ − *DF*_c_ map contoured at 0.34 e^−^ Å^−3^). Also shown are the positions of the selenium sites found in the bowl for each of the methionine mutants indicated, showing good agreement with the positions of the corresponding wild-type residues that were mutated. (*b*, *c*) Views of the hinge-binding site in the locked (*b*) and open (*c*) conformational states; in the ‘open’ state (*c*), the hinge residues from the ‘locked’ state βhingeH6.AP2 structure are superposed onto the ‘open’ structure and shown in grey. Adapted from Kelly *et al.* (2014 ▸).

In the case of the wild-type βhingeHis6.AP2, selenium sites corresponding to the ‘core’ methionines (*i.e.* methionines in the previously solved AP2 core) were found, including several that most likely represented alternative methionine conformers; no additional sites that might correspond to the C-terminal pair of methionines (Fig. 2 ▸ *f*) were found. The methionine point mutant βhingeHis6.AP2 complexes yielded similar results, except that in four of the mutants (Q619M, Q624M, D626M and L632M) a single selenium site distinct from the ‘core’ methionine positions was found in the bowl (Figs. 3 ▸ *d*, 3 ▸ *e*, 3 ▸ *f* and 3 ▸ *g*) close to the unmodelled difference density. A summary of crystallographic data for these mutants is presented in Table 2 ▸. In all cases the anomalous site within the bowl was the highest peak that could not be attributed to a methionine residue within the AP2 core structure. An example of the sites found by *Phaser-EP* over the course of several cycles of substructure completion is shown in Fig. 5 ▸. Since the sites present in the bowl could not be explained by any of the core methionines, we attributed them to the single methionine mutations introduced into each mutant. Based on our preliminary model for the buried hinge fragment, the spacing between these selenium sites was consistent with the spacing between the residues mutated to methionine in these mutants. This allowed us to fix the register and directionality of the hinge residues, showing that the clathrin-box motif is indeed buried in the core and thus inaccessible to clathrin in this conformation (Fig. 6 ▸ *a*). It is interesting to note that *JPred* secondary-structure prediction had suggested that residues Asp626–Leu631 form a short stretch of α-helix; based on our selenomethionine marker strategy, these residues indeed correspond to a short region that is α-helical.

For several mutants (L615M, V620M and L628M) no selenium sites were found in the hinge region. L615M lies outside the ordered region based on our subsequent model building (see below); in the case of V620M the overall difference electron density in the bowl is very poor. For the L628M mutant only one data set was collected and this did not reveal a selenium site in the hinge. One mutant, L631M, yielded a site in the hinge region at a location commensurate with the final assigned structure, but the *Z*-score of this site (∼5) was less than our cutoff value of 6 for new sites.

In a few cases, selenium sites were found at cysteines in the relatively rigid and well ordered σ2 subunit. We speculate that these are sites where selenocysteine has been incorporated in place of cysteine, either because of traces of selenocysteine present in our selenomethionine stock or perhaps owing to the salvage of selonocysteine from selenomethionine; however, unlike mammalian cells, there is no documented methionine-to-cysteine salvage pathway in *E. coli*.

The only anomalous scattering site that could not be attributed to selenomethionine or selenocysteine coincided with a small unmodelled region of difference electron density. We ascribed this to a chloride ion because of its electron-dense nature (the *B* factor of a water molecule placed at this position refined to an unrealistically low value) and because of the presence of two backbone N atoms and one O atom within ∼3.5 Å (corresponding to the first coordination sphere; Carugo, 2014 ▸). Chlorine has an *f*′′ of ∼0.3 electrons at the selenium edge, compared with ∼3.8 electrons for selenium, but we estimated the Se incorporation to be only ∼0.45 (Fig. 3 ▸). Thus, it is plausible that a well ordered chloride ion with an occupancy of one might cause an anomalous peak height of about a fifth the size of a selenium and thus be found as a weak site in our analysis.

The selenium sites found in the bowl of the four point mutants are ‘weak’ compared with the majority of sites attributable to core methionines. This is consistent with the notion of a partially buried hinge fragment, since tight binding would preclude rather than reduce clathrin binding to AP2. Since we were able to match four selenium sites with their expected positions based on our model, and given the lack of ‘spurious’ sites that could not be attributed to methionine residues (or in rare cases to cysteines or a halide ion), we were confident in our assignment of the hinge sequence.

## 6. Final model building, refinement and biological implications   

The model was refined by iterative rounds of rebuilding in *Coot* (Emsley *et al.*, 2010 ▸) and TLS and restrained refinement in *REFMAC*5. *MolProbity* (Chen *et al.*, 2010 ▸), accessed *via* the *PHENIX* interface (Echols *et al.*, 2012 ▸), and the validation tools within *Coot* were consulted throughout the refinement process. The final model had *R* and *R*_free_ residuals of 0.203 and 0.259, respectively, and good stereochemistry (r.m.s.d.s of 0.013 Å for bond lengths and 1.54° for bond angles; Table 1 ▸). In common with the original AP2 core structure (Collins *et al.*, 2002 ▸), the helical solenoid of the β2 trunk is followed by a stretch of extended peptide and a trio of short helices that pack against each other and against the β2 trunk; after Val582 the hinge becomes disordered. Our new structure (Fig. 6 ▸ *a*) reveals that after 35 disordered residues, the β2 hinge then loops back in towards the bowl of AP2, forming a short stretch of β-sheet with a loop between two helices of the α-subunit solenoid; there follows a turn and an α-helix that includes the first few residues of the clathrin-box motif before the electron density is lost.

**Figure 7:**
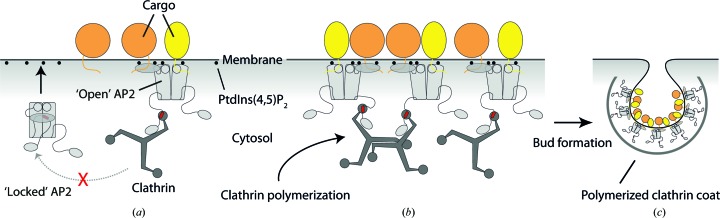
Model for endocytic pit initiation. (*a*) In the ‘locked’, cytosolic state, both clathrin and cargo binding by AP2 is autoinhibited. Recruitment to the plasma membrane (horizontal grey line) by interactions with PtdIns(4,5)P_2_ (black spheres) stimulates a conformational change in AP2 (light grey) that reveals both the binding sites for cargo-peptide motifs (yellow/orange lines) and the clathrin-binding epitope (red bar). Binding both clathrin and cargo stabilizes AP2 in the ‘open’ conformational state. (*b*) Once stabilized on the membrane, AP2 binds and recruits further clathrin (dark grey), stimulating clathrin polymerization. (*c*) Polymerized clathrin, with the assistance of other factors (Miller *et al.*, 2015 ▸), deforms the membrane to produce a curved clathrin-coated ‘bud’ that ultimately undergoes scission to produce a mature transport vesicle.

Alignment of the open AP2 conformation with our new extended AP2 locked conformation allowed us to propose a mechanism for the regulation of clathrin binding. The two structures were aligned on the rigid regions of structure proximal to the buried hinge fragment (residues 480–510 of the α subdomain; Figs. 6 ▸ *b* and 6 ▸ *c*). In the open conformation the entrance to the bowl from this side of the complex collapses, blocking the entry point of the hinge (Fig. 6 ▸ *c*). Thus, in the open conformation the β2 hinge must be released from the bowl, allowing clathrin to bind. This model (Fig. 7 ▸) explained our biochemical observation that clathrin recruitment and polymerization is stimulated by the binding of AP2 to a PtdIns(4,5)P_2_- and cargo-containing membrane.

Interestingly, the related clathrin adaptor AP1, which mediates trafficking between certain internal compartments (trans-Golgi network and endosomes), undergoes similar conformational rearrangements upon membrane localization, albeit driven by binding to the membrane-bound small GTPase Arf1 (Ren *et al.*, 2013 ▸) rather than to PtdIns(4,5)P_2_. The hinge of the AP1 β1 subunit (equivalent to β2 in AP2) contains a similar clathrin-box motif and flanking sequence (Kelly *et al.*, 2014 ▸). It remains to be seen whether or not clathrin recruitment by AP1 is regulated similarly to AP2.

## 7. Conclusions   

Our studies have shown that useful information can be obtained from partial selenomethionine-incorporation strategies when full incorporation is prohibited owing to problems with protein production. Although it was necessary to screen multiple crystals or sites on large crystals in order to obtain the best diffraction and anomalous signal, this is now a practical approach because of improvements in synchrotron beamlines and X-ray diffraction detectors that have dramatically increased the speed of data collection. Our crystallographic studies provided a structural framework to design biochemical experiments that elucidated how AP2 keeps its clathrin-binding motif hidden from clathrin until it is correctly localized at the plasma membrane and bound to cargo (Kelly *et al.*, 2014 ▸).

## Figures and Tables

**Table 1:** Crystallographic data for βhingeHis6.AP2 Values in parentheses are for the highest resolution bin.

Data collection	
Resolution range (Å)	97.37–2.79 (2.86–2.79)
Beamline	I03, DLS
No. of crystals	1
Wavelength (Å)	0.9393
*R*_merge_	0.073 (0.860)
*R*_meas_	0.076
*R*_p.i.m._ (within *I* ^+^/*I* ^−^)	0.023 (0.032)
Mean *I*/σ(*I*)	21.9 (3.2)
Completeness (%)	99.93 (100)
Multiplicity	10.9 (11.2)
Wilson *B* factor (Å^2^)	85.56
CC_1/2_	0.999 (0.859)
CC*	1.000 (0.961)
Total reflections	604922 (61719)
Unique reflections	55666 (5521)
Space group	*P*3_1_21
Unit-cell parameters (Å)	*a* = *b* = 121.3, *c* = 259.4
Refinement	
*R*/*R*_free_	0.2034/0.2594
No. of non-H atoms	13889
Average *B* factor (Å^2^)	83.8
Ramachandran favoured (%)	93.0
Ramachandran outliers (%)	1.0
R.m.s.d., bond lengths (Å)	0.013
R.m.s.d., bond angles (°)	1.54
Clashscore†	10.34

† As defined in Chen *et al.* (2010 ▸).

**Table 2:** Crystallographic data for selenomethionyl βhingeHis6.AP2 mutants Values in parentheses are for the highest resolution bin.

	Q619M SeMet	Q624M SeMet	D626M SeMet	L632M SeMet
Resolution range (Å)	60.8–3.1 (3.15–3.07)	66.9–3.2 (3.29–3.21)	64.5–3.2 (3.24–3.16)	86.2–3.3 (3.33–3.25)
Beamline	I03, DLS	I04-1, DLS	I02, DLS	I04-1, DLS
No. of crystals	2	1	2	1
Wavelength (Å)	0.9762	0.9795	0.9795	0.9173
*R*_merge_	0.155 (0.725)	0.113 (0.742)	0.127 (0.886)	0.134 (0.841)
*R*_meas_ (within *I* ^+^/*I* ^−^)	0.158 (0.157)	0.131 (0.125)	0.138 (0.131)	0.138 (0.137)
*R*_p.i.m._ (within *I* ^+^/*I* ^−^)	0.027 (0.036)	0.040 (0.053)	0.025 (0.032)	0.031 (0.042)
Mean *I*/σ(*I*)	31 (4.5)	15.5 (3.3)	29.9 (4.4)	21.7 (4.5)
Completeness (%)	100.0 (100.0)	99.8 (99.8)	100.0 (100.0)	100.0 (100.0)
Multiplicity	35 (28.1)	10.5 (11.2)	31.1 (22.5)	20.1 (21.5)
Anomalous completeness (%)	100.0 (100.0)	99.8 (99.8)	100.0 (100.0)	100.0 (100.0)
Anomalous multiplicity	18.3 (14.4)	5.5 (5.8)	16.3 (11.7)	10.6 (11.1)
Δ_anom_ correlation (half data sets)	0.408	0.348	0.808	0.135
Anomalous normal probability slope	1.114	1.211	1.470	1.058
Total reflections	1480736	389509	1200576	718549
Total unique reflections	42274	36952	38649	35814
